# Guanxinning Tablet Ameliorates Erectile Dysfunction in Diabetes Mellitus Rats with Involvement of PI3K/Akt/FOXO1 Signaling Pathway Modulation

**DOI:** 10.3390/biomedicines14081787

**Published:** 2026-08-07

**Authors:** Sheng Xin, Xiaming Liu, Jiaquan Mao, Bin Yang, Tao Wang, Xiaodong Song, Jihong Liu, Delin Ma, Wen Song

**Affiliations:** 1 Department of Urology, Tongji Hospital, Tongji Medical College, Huazhong University of Science and Technology, Wuhan 430030, China; 2 Hubei Provincial Clinical Research Center for Minimally Invasive Treatment of Urology, Wuhan 430071, China; 3 Department of Urology, Wuhan Central Hospital, Tongji Medical College, Huazhong University of Science and Technology, Wuhan 430030, China; 4 Department of Endocrinology, Tongji Hospital, Tongji Medical College, Huazhong University of Science and Technology, Wuhan 430030, China

**Keywords:** Guanxinning tablet, erectile dysfunction, diabetes mellitus, PI3K/Akt/FOXO1 pathway, apoptosis

## Abstract

**Background/Objectives**: Diabetes mellitus-induced erectile dysfunction (DMED) is characterized by severe endothelial and smooth-muscle dysfunction and reduced responsiveness to conventional therapy. Guanxinning tablet (GXN), a traditional Chinese medicine compound containing *Salvia miltiorrhiza* (Danshen) and *Ligusticum stratum* (Chuanxiong), has vascular-protective and anti-oxidative properties, but its effect on DMED remains unclear. This study evaluated whether GXN improves erectile function in a streptozotocin (STZ)-induced type 1 diabetic rat model and explored the possible involvement of PI3K/Akt/FOXO1 signaling. **Methods**: STZ-induced diabetic rats with ED were treated with GXN. Artery pressure (AP) and intracavernous pressure (ICP) were measured to evaluate erectile dysfunction. Western blots, immunohistochemistry, immunofluorescence, biochemical assays, TUNEL staining, RNA-seq, and in vitro HCMEC assays were performed to evaluate erectile effector cell function, oxidative stress, fibrosis, apoptosis, and PI3K/Akt/FOXO1-associated changes. **Results**: GXN improved erectile function in DMED rats and was associated with improved endothelial and smooth-muscle-related markers, reduced oxidative stress, attenuated fibrosis, and decreased apoptosis. RNA-seq and protein validation suggested that GXN treatment was associated with activation of PI3K/Akt/FOXO1-related signaling. In HCMECs, LY294002 (a PI3K inhibitor) partially reversed the GXN-associated improvement in cell viability and apoptosis-related markers. **Conclusions**: GXN ameliorated erectile dysfunction and tissue injury in STZ-induced type 1 diabetic rats, which were associated with PI3K/Akt/FOXO1 signaling. These findings support the potential of GXN as a traditional Chinese medicine compound for treating DMED.

## 1. Introduction

Diabetes mellitus (DM) is a global chronic disease that adversely affects multiple organs, notably the cardiovascular system, the nervous system, and the endocrine system [1]. The pathogenesis of erectile dysfunction (ED), which is characterized by insufficient hardness or inability to maintain penile erection, is multifaceted, involving vascular disease, neuropathy, hormone imbalance, and psychological factors [2]. DM-induced ED (DMED) is a prevalent complication among male DM patients [3]. This form of ED not only presents with more severe symptoms and reduced efficacy of traditional treatments [4] but may also be a precursor to cardiovascular disease [5], warranting clinical attention.

The systemic chronic inflammatory environment caused by diabetes is closely related to the mechanisms of vascular injury, oxidative stress, fibrosis and apoptosis, which lead to DMED [6,7,8]. The PI3K/Akt/FOXO1 pathway is crucial in cell survival, metabolism, proliferation, oxidative-stress responses, and apoptosis [9,10]. PI3K/Akt signaling can regulate insulin-mediated glucose uptake and utilization, particularly by promoting the expression and activity of nitric oxide (NO) synthase, thereby affecting vasodilation, which is crucial for maintaining normal erectile function [11,12]. FOXO1 is a downstream transcription factor that is negatively regulated by Akt-mediated phosphorylation [13], which subsequently influences glucose metabolism and oxidative stress responses [14]. PI3K/Akt/FOXO1 is the predominant signal for lessening the level of blood glucose [15]. The activation of the PI3K/Akt/FOXO1 pathway can be a potential way to reduce oxidative stress and inflammation in the liver of diabetes rats and regulate liver regeneration [16]. Importantly, it is demonstrated that modulation of PI3K/Akt/FOXO signaling may repair cavernous nerves and improve neurogenic ED [17]. Therefore, we hypothesize that the disruption of PI3K/Akt/FOXO signaling contributes to the progression of DMED and that its activation may be associated with restoring damaged erectile effector cell function in the corpus cavernosum.

In recent years, natural products and their active compounds have emerged as a promising alternative therapeutic strategy for the management of diabetes mellitus and its associated complications owing to their multi-target effects and low toxicity [18,19,20]. Guanxinning tablet (GXN) is a traditional patent medicine composed of various Chinese herbal ingredients, primarily including Salviae Miltiorrhizae Radix et *Salvia miltiorrhiza* (Danshen) and *Ligusticum stratum* (Chuanxiong). Recent scientific studies have shown that GXN and its active ingredients can treat DM and its complications through a variety of mechanisms, including improving blood microcirculation, reducing oxidative stress responses, regulating immune responses, etc. [21,22]. Xu Z et al. confirmed that the efficacy of Danshen in improving renal injury in diabetes is related to the PI3K/Akt/FOXO pathway [23]. Additionally, Chuanxiong has been shown to promote angiogenesis via the PI3K/Akt/Ras/MAPK pathway [24]. Although GXN and its components have been studied in cardiovascular and diabetic complications, GXN’s effect on DMED and its association with PI3K/Akt/FOXO1-related anti-apoptotic and vascular-protective signaling had not been defined. However, these observations provide the rationale for testing GXN in DMED, a disease in which endothelial dysfunction, oxidative stress, apoptosis, and fibrosis jointly impair erectile function.

In this study, we used an STZ-induced type 1 diabetes rat model to evaluate the therapeutic effect of GXN on DMED. We assessed erectile function, endothelial and smooth-muscle-related pathways, oxidative stress, fibrosis, apoptosis, and transcriptomic changes. We further used human cerebral microvascular endothelial cells (HCMECs) for in vitro high-glucose and PI3K-inhibition validation. This design allowed us to evaluate the protective phenotype of GXN and to explore whether PI3K/Akt/FOXO1 signaling is associated with its effect.

## 2. Materials and Methods

### 2.1. Animals

The in vivo design is shown in Supplementary Figure S1. The thirty male Sprague–Dawley (SD) rats involved in this study were 6–8 weeks old and underwent at least one week of environmental adaptation before undergoing the experiment. To construct the DMED model, we followed the previous protocol [8,25], which involved intraperitoneal injection of a single dose (60 mg/kg) of a 1% streptozotocin (STZ, Sigma-Aldrich, Saint Louis, MO, USA) solution into rats. After injection, rats whose abdominal blood glucose exceeded 16.7 mmol/L for three consecutive days were considered successful DM models. In the 8th week, apomorphine (APO) experiments were conducted on DM rats, and if there was no glans exposure or penis erection, ED modeling was successful. Based on the normalization of standard body surface area and previous studies [26,27], GXN is administered by gavage for 4 weeks at a dose of 1200 mg/kg/d, with equal doses of corn oil used as the control. Each group contained 10 rats for functional and molecular biology testing at week 12.

The entire experimental process strictly followed the welfare and usage standards of experimental animals set out by the Institutional Animal Care and Use Committee (IACUC) of Tongji Medical College of Huazhong University of Science and Technology (TJH-202205016), ensuring that all animals received reasonable care and treatment.

### 2.2. Evaluation of Erectile Dysfunction

Following anesthesia with an intraperitoneal injection of 20 mg/kg of the 0.4% pentobarbital sodium solution, the BL-420F physiological signal acquisition system (Techman Soft, Chengdu, China) was employed to catheterize the carotid artery catheter, exposing the cavernous nerves on both sides of the prostate and providing electrical stimulation (15 Hz, 5 V, 1 min). The arterial pressure (AP) and intracavernous pressure (ICP) were monitored and recorded. After the detection step was completed, the penis was removed and stored according to the requirements of subsequent experiments.

### 2.3. Cell Culture

HCMECs (Miao Ling Biotechnology, Wuhan, China) were used as an accessible human microvascular endothelial-cell model to validate high-glucose-induced endothelial injury and pathway-associated responses in vitro. HCMECs were cultivated in ECM (Warner, Wuhan, China) supplemented with 10% FBS, 1% pen/step, and 1% ECGS under conditions of 5% CO2 and 37 °C. Referring to previous studies [17,28,29], we induced HCMECs using glucose (30 mM; Sigma-Aldrich, Saint Louis, MO, USA), GXN (1 mg/mL), and LY294002 (10 μM; a PI3K inhibitor, MedChemExpress, NJ, USA) to establish four groups: the low-glucose (LG), high-glucose (HG), HG + GXN, and HG + GXN + LY groups.

### 2.4. Western Blot Analysis

According to the protocol outlined in the previous literature [30]. Western blot (WB) analysis was conducted to confirm the expression level of the target protein. We incubated target protein bands with primary antibodies (detailed information provided in Supplementary Table S1), goat antibodies coupled with horseradish peroxidase (HRP), and Western ECL substrates (Biossci, Wuhan, China) sequentially. Detection was performed using the ChemiDoc™ MP imaging system (Bio-Rad Laboratories, Hercules, CA, USA).

### 2.5. Histologic Evaluation

#### 2.5.1. Immunohistochemistry (IHC)

For IHC, paraffin-embedded penile tissue was cut into 5 um sections, dewaxed, hydrated, and subjected to antigen retrieval. After blocking, sections were incubated with primary antibodies (Supplementary Table S1), HRP-conjugated secondary antibodies, the DAB colorimetric solution, and hematoxylin. Images were acquired using an optical microscope. Quantification was performed using ImageJ (Fiji ImageJ 1.54).

#### 2.5.2. Fluorescence Staining

Immunofluorescence (IF) was performed using 5 µm paraffin sections, followed by infiltration and blocking, and sections were incubated with primary antibodies (Supplementary Table S1) and fluorescent-labeled secondary antibodies. After staining the nucleus with DAPI and sealing it, images were observed and captured using the fluorescence microscope.

Reactive oxygen species (ROS) was detected in frozen penile sections using Dihydroethidium (DHE; red fluorescence; Beyotime Biotechnology, Shanghai, China) fluorescent probes according to the instructions of the reagent kit.

#### 2.5.3. Masson’s Trichrome Staining

The paraffin sections of the penis were stained following the recommended protocol of the reagent kit (Servicebio, Wuhan, China). Collagen fibers in the corpus cavernosum appeared blue, while smooth muscle fibers appeared red. The ratio of smooth muscle to collagen fiber area reflects the degree of fibrosis in the corpus cavernosum.

### 2.6. Detection of Oxidative Stress and Endothelial Function

Oxidative stress was comprehensively assessed according to the experimental protocol recommended by the reagent kit by measuring malondialdehyde (MDA; Beyotime Biotechnology, Shanghai, China) and superoxide dismutase (SOD; Beyotime Biotechnology, Shanghai, China) levels to evaluate the level of lipid peroxidation and antioxidant product activity.

The detection kits were used to detect NO (Beyotime Biotechnology, Shanghai, China) and cyclic guanosine monophosphate (cGMP; Mbbiology, Yancheng, Jiangsu, China) in tissue lysates and to evaluate endothelial function.

### 2.7. RNA-Sequencing (RNA-Seq)

RNA was extracted from the corpus cavernosum of the penis using Trizol (15596018, Thermo Fisher Scientific, Waltham, NY, USA), and its concentration was determined via a nanophotometer (IMPLEN, CA, USA). The extracted RNA was used to construct a sequencing library using Illumina’s NEBNext UltraTM RNA library preparation kit (NEB, Ipswich, MA, USA). After the sequencing data was generated, preliminary quality control was carried out using fastp (Version 0.23.2) [31], including splicing and filtering low-quality read segments, and the alignment tool HISAT2 (Version 2.2.1) [32] to accurately align read segments to the reference genome or transcriptome. Subsequently, featureCounts (Version v2.0.1) [33] was used to quantify the expression levels of genes, and DESeq2 (Version 1.20) [34] was used to analyze differential expression under different conditions. Differential expression genes (DEGs) were defined as |log2FoldChange| > 1 with adjusted *p*-values < 0.05. The functional enrichment analysis was implemented via clusterProfiler (Version 4.2.0) [35].

### 2.8. Detection of Apoptosis

Terminal deoxynucleotidyl transferase-mediated dUTP nick end labeling (TUNEL) staining was performed according to the protocol of the assay kit (Servicebio, Wuhan, China) to detect apoptosis levels in the corpus cavernosum. Brown staining of the cell nucleus indicates apoptosis, while blue staining indicates normal cells. The apoptosis index, representing the percentage of apoptotic cells among all cells, reflects the level of apoptosis in the tissue.

Cells were collected after treatment and stained according to the manufacturer’s instructions. We used a flow cytometer (Beckman, Brea, CA, USA) to detect the cell apoptosis rate, with quadrant gates set as Q1 for necrotic cells, Q2 for late apoptotic cells, Q3 for live cells, Q4 for early apoptotic cells. Total apoptosis rate = (proportion of early apoptotic cells + proportion of late apoptotic cells) × 100%.

### 2.9. Statistical Analysis

The GraphPad Prism 9 software (GraphPad software Inc., SD, USA) supported all data processing in this experiment and reflected statistical results with the mean ± standard deviation. Normality was assessed using the Shapiro–Wilk test, and homogeneity of variance was assessed using the Bartlett test. For comparisons between two groups, a *t*-test was used; for single-time-point comparisons among more than two groups, one-way ANOVA followed by Tukey’s post hoc test was applied; for repeated measurements of body weight and fasting blood glucose over time, we used two-way ANOVA and performed Dunnett’s multiple comparison test. *p* < 0.05 was considered statistically significant.

## 3. Results

### 3.1. GXN Enhanced Erectile Function in DMED Rats

**Figure 1:**
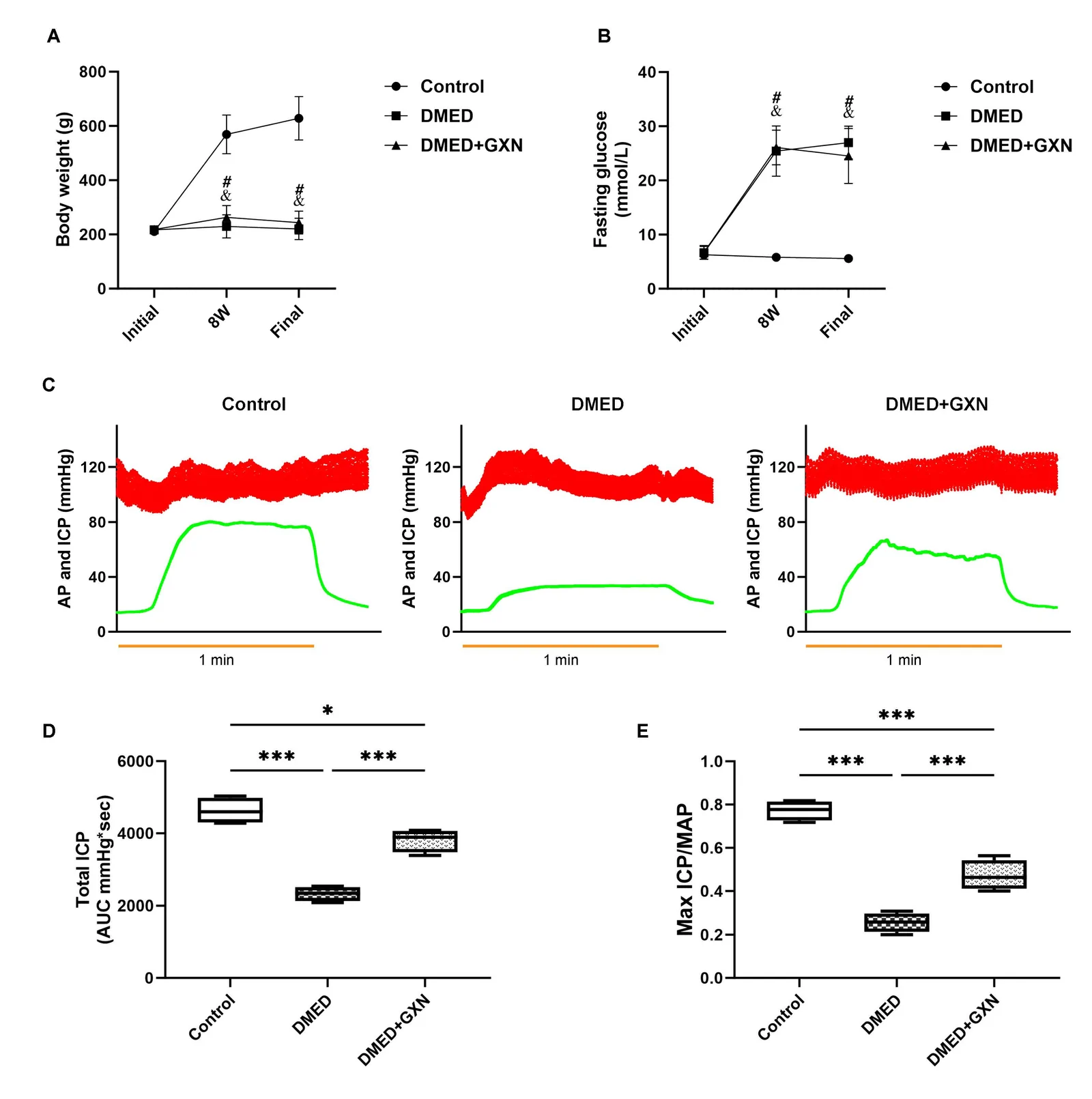
Physiological indicators of the rats during the experiment. Line chart of body weight (**A**) and fasting blood glucose fluctuations (**B**) in each group (n = 10). (**C**) Representative AP and ICP waveforms of each group during cavernous nerve electrical stimulation. Semi-quantitative results of the maximum ICP/MAP (**D**) and AUC of ICP (**E**) for each group (n = 4). &: *p* < 0.05 when comparing the DMED group versus the control group, #: *p* > 0.05 when comparing the DMED + GXN group versus the DMED group, *: *p* < 0.05, ***: *p* < 0.001.

Among the 36 rats that received STZ injection, 33 successfully contracted diabetes. At week 8, excluding weak or dead rats, 23 of the remaining 28 diabetic rats had an ED phenotype, 20 of which were included in the follow-up experiment. Body weight and fasting blood glucose were monitored throughout the experiment (Figure 1A,B). It is shown that DMED rats had reduced body weight and increased fasting blood glucose compared with control rats. GXN treatment did not significantly alter body weight or fasting glucose trends compared with untreated DMED rats.

In physiological assessments, the MAP of rats remained unaffected by blood glucose levels, stabilizing at approximately 100 mmHg (Figure 1C). The representative ICP waveforms revealed that the peak ICP in DMED rats was lower than that of normal rats; however, GXN supplementation increased the peak. Semi-quantitative results also confirmed that the ratio of maximum ICP to MAP and the area under the curve (AUC) of ICP in DMED rats were significantly reduced compared to normal rats, and these two indicators were improved after supplementing with GXN (Figure 1D,E). These data indicate that GXN effectively ameliorated erectile function impaired by hyperglycemia.

### 3.2. GXN Was Associated with Improved eNOS-NO-cGMP and RhoA-ROCK Pathway Markers In Vivo

**Figure 2:**
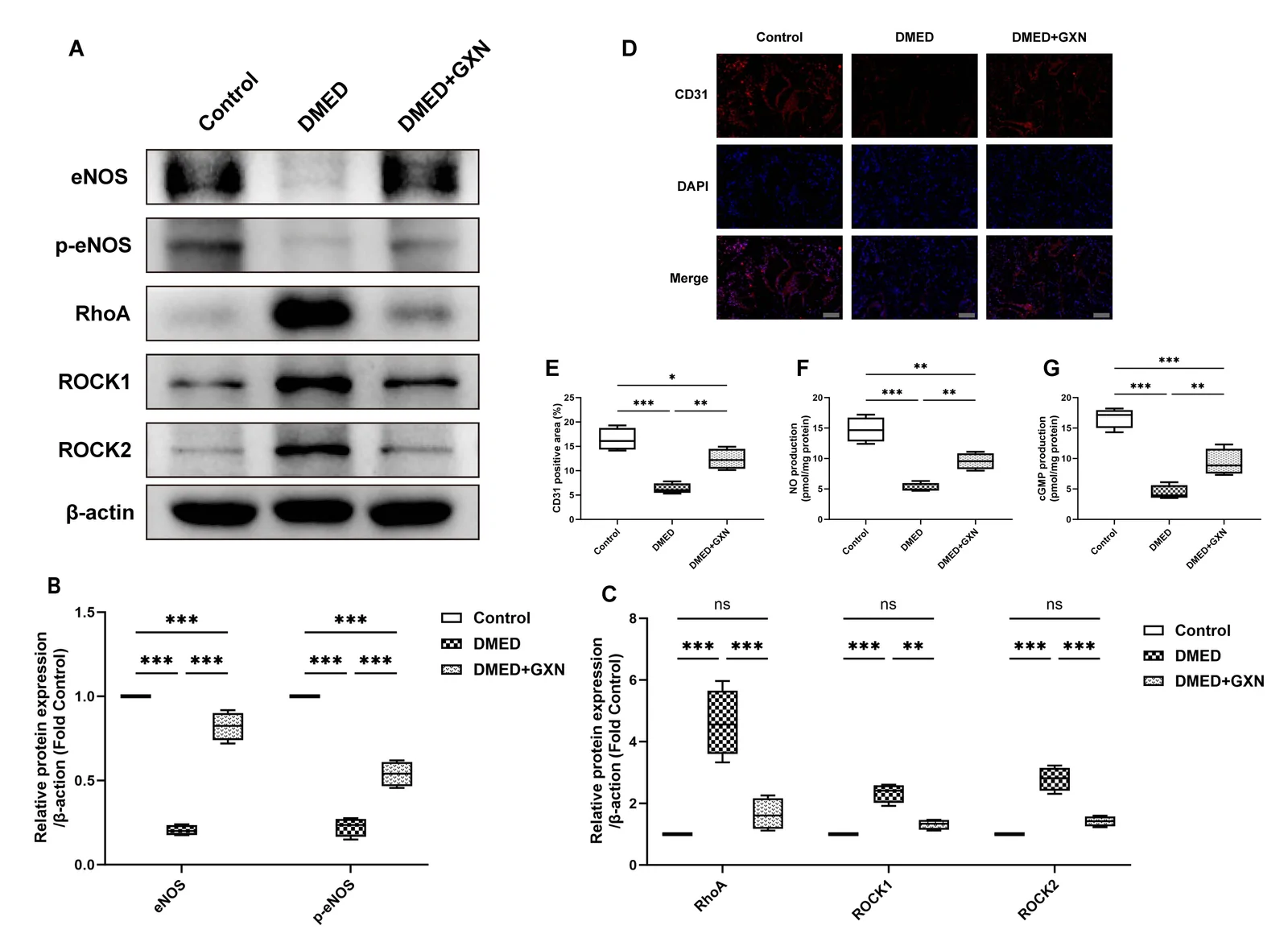
Stimulating activity of GXN on the erectile pathway. (**A**) Representative images of eNOS, p-eNOS, RhoA, ROCK1, and ROCK2 in WB analysis. (**B**) Semi-quantitative results of WB bands (eNOS and p-eNOS, n = 4). (**C**) Semi-quantitative results of WB bands (RhoA, ROCK1, and ROCK2, n = 4). (**D**) Representative images of CD31 expression in IF (×200, bars = 100 µm). (**E**) Semi-quantitative result of CD31 in IF. NO (**F**) and cGMP (**G**) contents of each group. ns: no significance, *: *p* < 0.05, **: *p* < 0.01, ***: *p* < 0.001.

ED is typically caused by impaired vasodilation resulting from dysfunction in endothelial and smooth muscle cells. Compared to the control group, eNOS and its phosphorylated form generated by endothelial cells in the corpus cavernosum of DMED rats significantly decreased (Figure 2A,B), leading to a decrease in NO and cGMP content (Figure 2E,F). Additionally, the IF-positive area for CD31 (a surface marker of endothelial cells) in DMED rats was lower compared to that in normal rats (Figure 2C,D). However, the impaired function of endothelial cells significantly recovered after GXN supplementation.

The puissant relaxation function of smooth muscles depends on the inhibition of the RhoA/ROCK pathway [36]. Our findings indicated that the expression of RhoA, ROCK1, and ROCK2 in DMED rats was elevated (Figure 2A,G). Furthermore, GXN effectively inhibited the RhoA/ROCK pathway, suggesting the restoration of impaired smooth muscle function. Based on these results, it can be inferred that the dysfunction of the corpus cavernosum caused by hyperglycemia primarily arose from damage to the function of effector cells, and GXN demonstrated a significant alleviating effect on this dysfunction.

### 3.3. GXN Remitted Oxidative Stress in the Corpus Cavernosum

**Figure 3:**
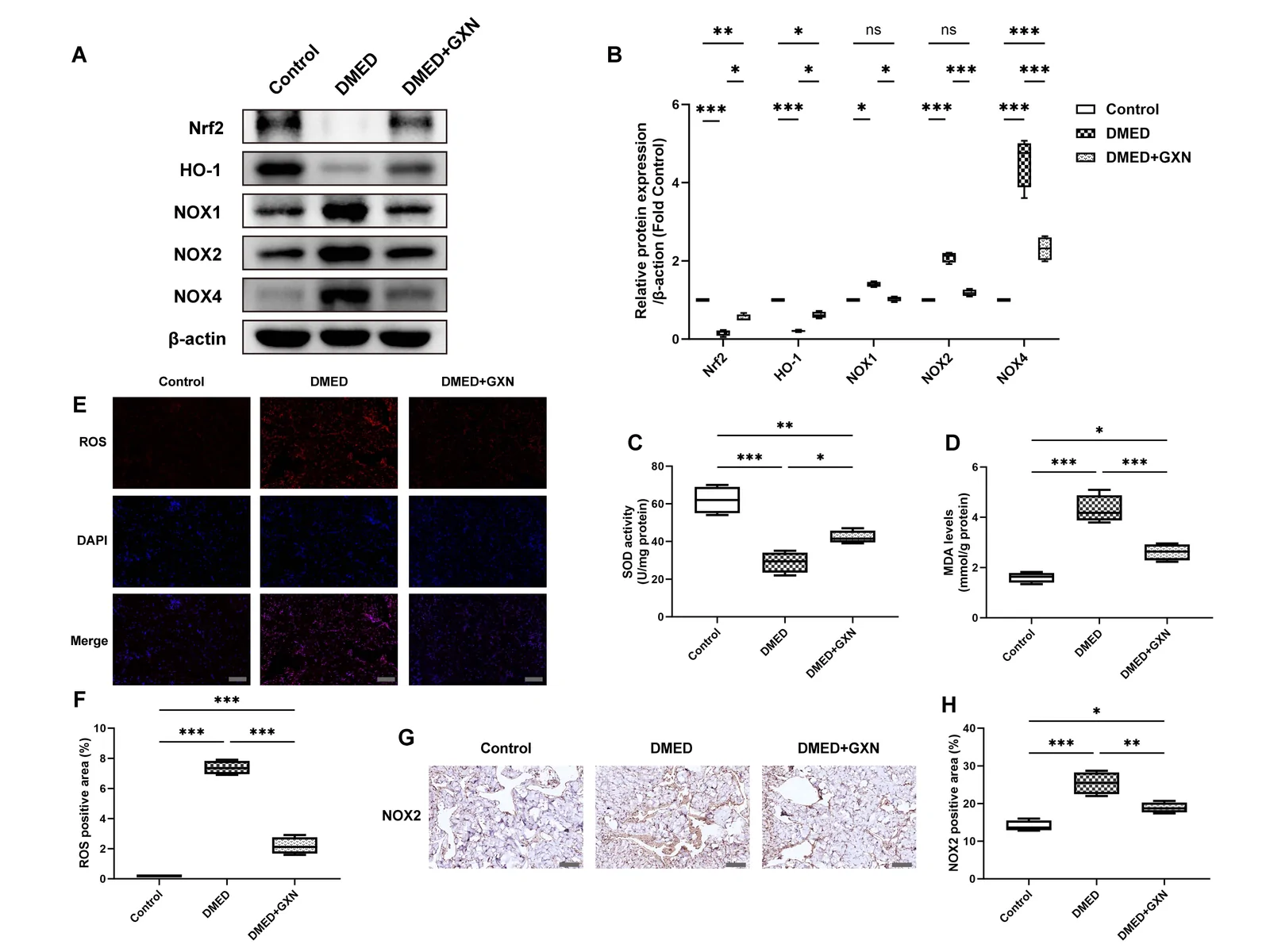
Reducing effect of GXN on oxidative stress. (**A**) Representative images of NADPH oxidases (NOX1, 2, and 4) and the Nrf2/HO-1 pathway in WB. (**B**) Semi-quantitative results of WB bands (n = 4). SOD (**C**) and MDA (**D**) activities of each group (n = 4). Representative images (**E**) and semi-quantitative results (**F**) of the ROS of each group (×200, bars = 100 µm, n = 4). (**G**) Representative images of NOX2 in IHC (×200, bars = 100 µm). (**H**) Semi-quantitative analysis of NOX2 expression in IHC (n = 4). ns: no significance, *: *p* < 0.05, **: *p* < 0.01, ***: *p* < 0.001.

Hyperglycemia is frequently associated with the activation of oxidative stress and is closely linked to pathological states such as inflammation and cell inactivation [37]. WB analysis revealed that the expression of reduced nicotinamide adenine dinucleotide phosphate (NADPH) oxidase (NOX1, 2, and 4), the primary source of ROS in vivo, was activated by hyperglycemia but decreased following GXN supplementation (Figure 3A,B), a finding supported by IHC results for NOX2 (Figure 3G,H).

Subsequent detection using the DHE fluorescence probe indicated that the ROS-positive area in the corpus cavernosum of DMED rats was much higher than that in normal rats, with a reduction observed in GXN-treated rats (Figure 3E,F). Moreover, the Nrf2/HO-1 antioxidant pathway was inhibited by hyperglycemia and activated by GXN (Figure 3A,B). SOD is also one of the antioxidant elements, while MDA reflects the level of lipid peroxidation. The results demonstrated decreased SOD activity and elevated MDA levels in the DMED group, both of which were reversed by GXN treatment (Figure 3C,D).

### 3.4. GXN Reversed Fibrosis in the Corpus Cavernosum

**Figure 4:**
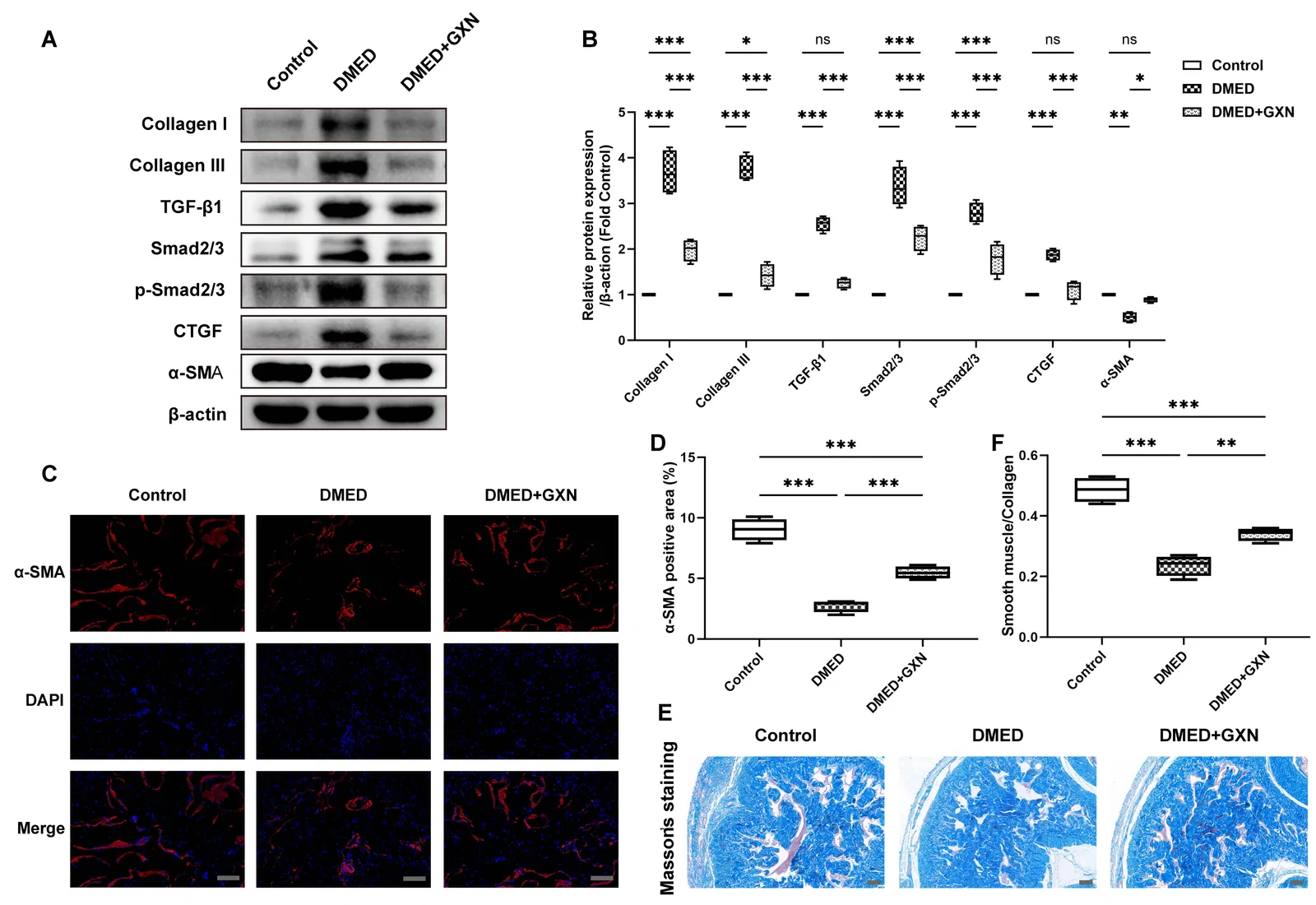
Reversion of GXN on cavernous fibrosis. (**A**) Representative images of fibrotic proteins and α-SMA in WB. (**B**) Semi-quantitative results of WB bands (n = 4). (**C**) Representative IF results of α-SMA (×200, bars = 100 µm). (**D**) Semi-quantitative expression of α-SMA in IF (n = 4). (**E**) Masson’s trichrome staining of each group (×100, bars = 100 µm). (**F**) Ratio of smooth muscle to collagen area in each group (n = 4). ns: no significance, *: *p* < 0.05, **: *p* < 0.01, ***: *p* < 0.001.

Corpus cavernosum smooth muscle fibrosis is characterized by an increase in the synthesis of collagen and connective tissue proteins by the vascular smooth muscle of the corpus cavernosum and is accompanied by a decrease in smooth muscle mass. WB analysis revealed that the expression of collagen I and III, along with pro-fibrotic factors (TGF-β1, Smad2/3, p-Smad2/3, CTGF), was significantly increased in the corpus cavernosum of DMED rats compared to normal rats, while the expression of alpha smooth muscle actin (α-SMA) was decreased (Figure 4A,B). IF results also confirmed a reduction in the area of α-SMA within the corpus cavernosum of DMED rats (Figure 4C,D). After supplementing with GXN, these indices, which were indicative of increased corpus cavernosum fibrosis, were reversed. Masson’s trichrome staining, which visualizes collagen fibers as blue and smooth muscles as red, quantifies the degree of tissue fibrosis by the ratio of collagen fibers to smooth muscle (Figure 4E,F). This analysis revealed a significant increase in corpus cavernosum fibrosis in DMED rats, which was ameliorated by GXN treatment.

### 3.5. GXN-Enhanced Erectile Function Was Associated with PI3K/Akt/FOXO1 Pathway Activation

**Figure 5:**
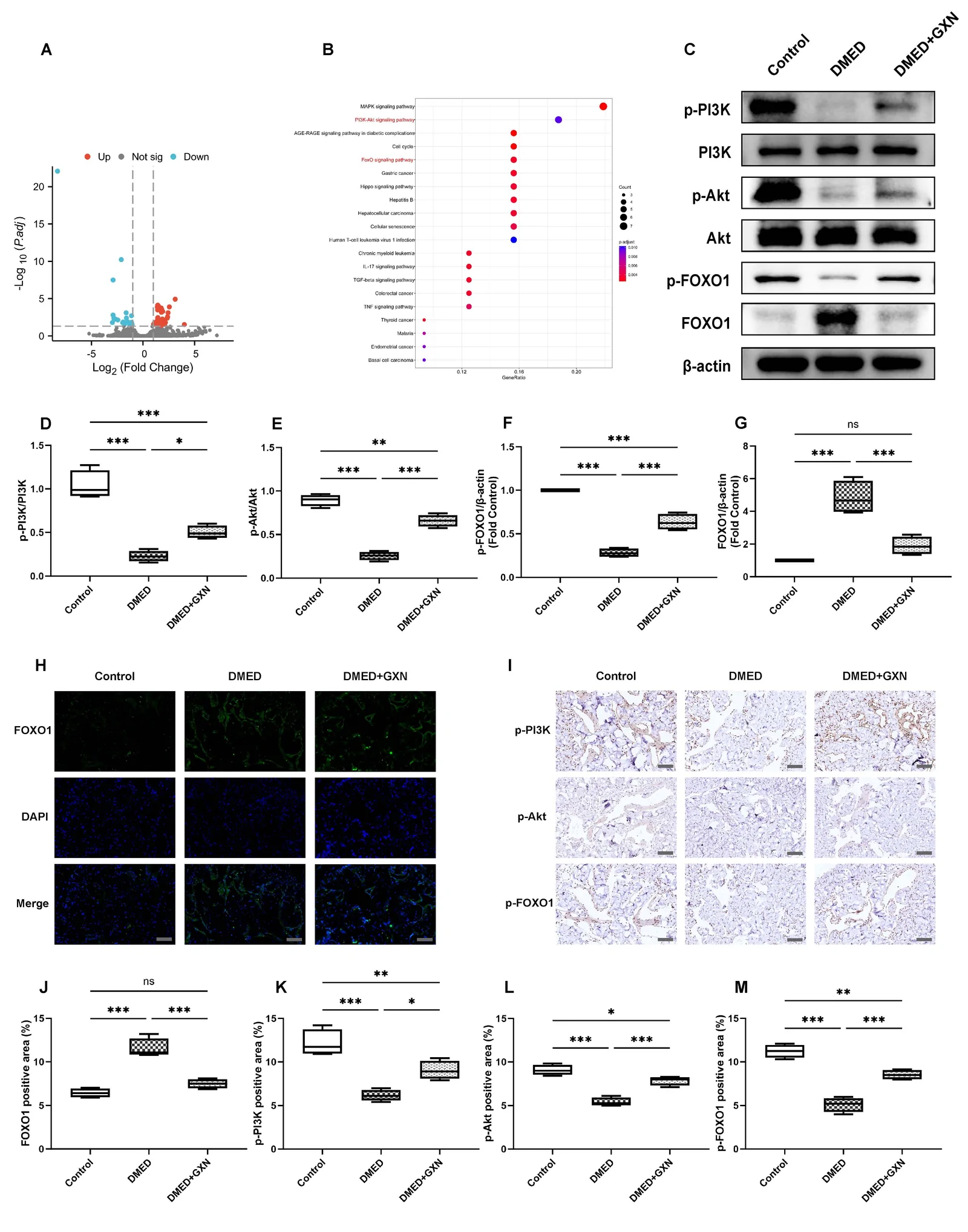
Effect of GXN on the PI3K/Akt/FOXO1 pathway in DMED rats. (**A**) Volcano diagram of DEGs after supplementing with GXN. (**B**) Differential enriched pathways of DEGs. (**C**) Representative WB images of PI3K/Akt/FOXO1 expression. (**D**–**G**) Semi-quantitative results of WB bands (n = 4). (**H**) Representative IF images of FOXO1 (×200, bars = 100 µm). (**I**) Representative IHC images of p-PI3K, p-Akt, and p-FOXO1 (×200, bars = 100 µm). (**J**) Semi-quantitative results of FOXO1 in IF (n = 4).(**K**–**M**) Semi-quantitative results of IHC (n = 4). ns: no significance, *: *p* < 0.05, **: *p* < 0.01, ***: *p* < 0.001.

To explore molecular pathways associated with the protective effects of GXN in DMED rats, we performed high-throughput sequencing on the DMED and DMED + GXN groups and conducted pathway enrichment analysis on DEGs (Figure 5A,B and Supplementary Figure S2). The enriched pathways were associated with cell regeneration and included the PI3K/Akt and FOXO-related pathways [38]. The PI3K/Akt pathway is an upstream negative regulatory pathway of FOXO1 [39]. Notably, the activation of the PI3K/Akt/FOXO pathway is used to repair neurogenic ED [17]. These findings suggest that PI3K/Akt/FOXO1 signaling is associated with the GXN response in DMED rats.

Therefore, we assessed the activation of the PI3K/Akt/FOXO1 pathway in each group. Hyperglycemia was found to reduce the phosphorylation levels of PI3K, Akt, and FOXO1, which partially recovered following GXN supplementation (Figure 5C–F). This finding was further corroborated by IHC results (Figure 5J–M). Additionally, the expression of FOXO1 was activated in DMED and inhibited after GXN treatment (Figure 5H,I).

### 3.6. GXN Ameliorated Apoptosis in the Corpus Cavernosum

**Figure 6:**
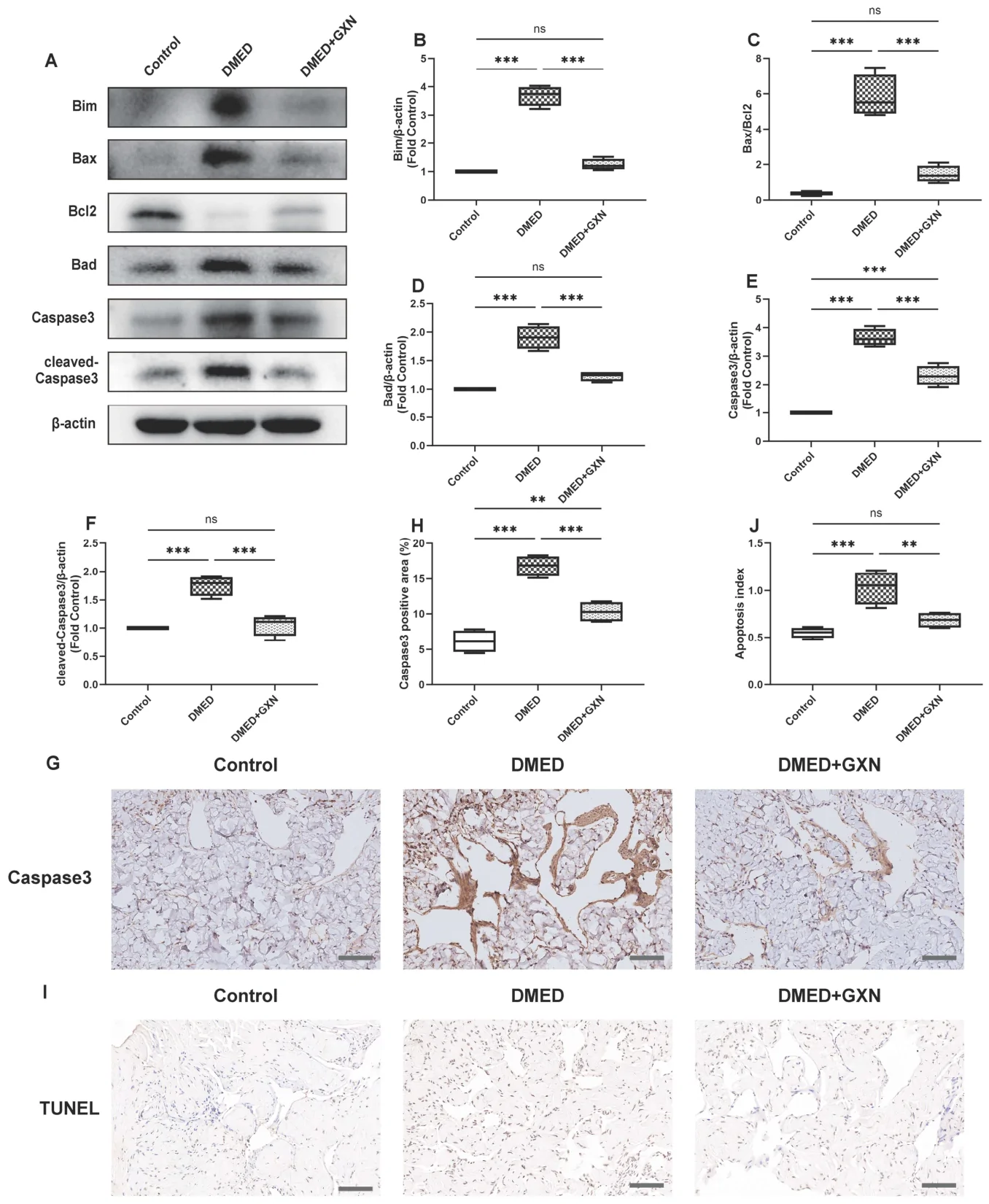
Inhibitory effects of GXN on apoptosis in DMED rats. (**A**) Representative WB images of apoptosis-related protein expression. (**B**–**F**) Semi-quantitative results of WB bands (n = 4). (**G**) Representative IHC images of Caspase3 (×200, bars = 100 µm). (**H**) Semi-quantitative results of Caspase3 in IHC (n = 4). (**I**) TUNEL staining images for each group (×200, bars = 100 µm). (**J**) Apoptosis index results for each group (n = 4). ns: no significance, **: *p* < 0.01, ***: *p* < 0.001.

Under specific cellular environments and signal stimuli, FOXO1 can activate a series of downstream genes, such as Bim and FasL, which promote the activation of apoptosis pathways [40]. In our study, we observed that several pro-apoptotic factors, including Bim, Bax, Bad, Caspase3, and cleaved-Caspase3, were upregulated in the DMED group (Figure 6A–F). In contrast, the anti-apoptotic factor Bcl-2 was downregulated, and GXN reversed the dysregulation of these proteins. Furthermore, the IHC analysis of Caspase3 corroborated the WB results (Figure 6G,H). TUNEL testing indicated that the DMED group exhibited the highest apoptosis index, which was significantly reduced following GXN supplementation (Figure 6I,J).

### 3.7. GXN Inhibited Apoptosis by Activating the PI3K/Akt/FOXO1 Pathway In Vitro

**Figure 7:**
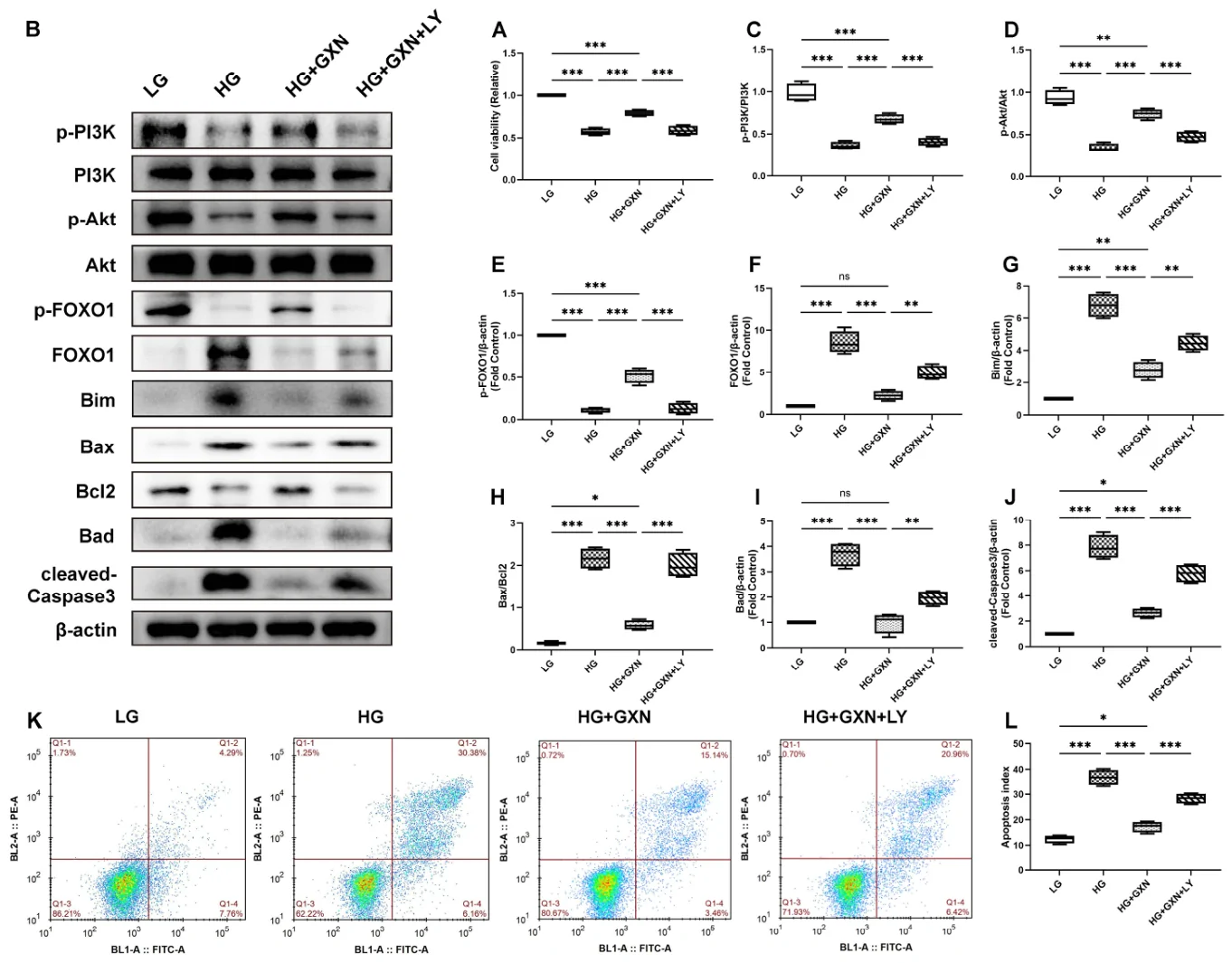
Promotion of the PI3K/Akt/FOXO1 pathway and inhibition of apoptosis by GXN in HCMECs. (**A**) Cell viability evaluation by CCK-8 (n = 4). (**B**) Representative WB images of the PI3K/Akt/FOXO1 pathway and apoptosis-related protein expression. (**C**–**J**) Semi-quantitative results of WB bands (n = 4). Flow cytometry images (**K**) and apoptosis rates (**L**) for each group (n = 4). ns: no significance, *: *p* < 0.05, **: *p* < 0.01, ***: *p* < 0.001.

In vitro experiments with HCMECs demonstrated that the cell viability of HCMECs was inhibited by HG and restored after supplementation with GXN, while LY294002 re-inhibited cell viability (Figure 7A).

The phosphorylation levels of PI3K, Akt, and FOXO1 were significantly reduced following HG induction. However, GXN supplementation effectively restored phosphorylation levels, an effect that was counteracted by the PI3K inhibitor LY294002 (Figure 7B–F).

Additionally, the expression of Bim, Bax, Bad, Caspase3, and cleaved-Caspase3 was increased in the HG group, while Bcl-2 expression was decreased. These changes were reversed in the GXN group (Figure 7B,G–J). The inhibitory effect of GXN on apoptosis was further diminished by LY294002, which is consistent with the observed trends in apoptosis rates measured by flow cytometry (Figure 7K,L).

## 4. Discussion

DMED is a frequent complication of diabetes and is characterized by endothelial dysfunction, impaired smooth-muscle relaxation, oxidative stress, fibrosis, apoptosis, and reduced responsiveness to conventional treatments [41]. GXN has been used clinically for cardiovascular conditions and has shown vascular-protective potential in previous experimental studies [42,43,44]. In the present study, GXN improved erectile function in STZ-induced type 1 diabetic rats and was associated with improved endothelial and smooth-muscle-related markers, reduced oxidative stress, attenuated fibrosis, and decreased apoptosis. These findings extend previous work on GXN by suggesting a potential therapeutic effect in DMED while also indicating that the PI3K/Akt/FOXO1 pathway may be involved in the response.

PI3K/Akt/FOXO1 signaling regulates cellular survival, metabolism, oxidative-stress responses, and apoptosis. PI3K activation generates PIP3, which contributes to Akt phosphorylation and downstream regulation of FOXO transcription factors [45]. Previous studies in DMED have confirmed the role of the PI3K/Akt pathway in regulating the eNOS/NO/cGMP pathway to enhance endothelial function [28], which was corroborated by the findings in DMED rats and HCMECs treated with GXN. In DMED, the apoptosis rate of erectile effector cells in the corpus cavernosum was significantly elevated [30], and FOXO1 has been implicated in the apoptosis of various cardiovascular diseases [46,47]. In this study, GXN treatment was associated with increased phosphorylation of PI3K, Akt, and FOXO1 and reduced expression of several apoptosis-related markers in vivo. In HCMECs, LY294002 partially reversed the GXN-associated changes in cell viability and apoptosis-related proteins. These data support pathway involvement. However, because LY294002 was used only in vitro and no in vivo pathway blockade or FOXO1 knockdown/overexpression was performed, the present data should not be interpreted as definitive proof that GXN acts exclusively through PI3K/Akt/FOXO1 signaling.

The corpus cavernosum depends on coordinated endothelial and smooth-muscle function. Endothelial NO production promotes cGMP synthesis in smooth muscle cells, resulting in reduced intracellular calcium levels, smooth-muscle relaxation, and increased cavernous blood filling [48]. In contrast, the RhoA/ROCK pathway plays a crucial role in regulating the function of vascular smooth muscle cells, particularly in maintaining vascular tension, vasoconstriction, and blood pressure regulation [36]. Activation of ROCK leads to increased phosphorylation of myosin light chains, promoting smooth muscle cell contraction. Notably, this pathway is also involved in the proliferation, migration, and apoptosis of vascular smooth muscle cells, contributing to vascular remodeling [49]. Our results showed that DMED reduced eNOS phosphorylation and NO/cGMP levels and increased RhoA/ROCK pathway markers, whereas GXN treatment was associated with partial recovery of these indices. These findings suggest that GXN may improve erectile function not only through apoptosis-related signaling but also by supporting endothelial-dependent relaxation and limiting excessive contractile signaling.

The pathogenesis of ED is complex, with oxidative stress playing a significant role, often manifesting as an excessive accumulation of ROS that adversely affects cell function and survival [50]. The primary sources of ROS are NADPH oxidase and the modulation of antioxidant response elements (AREs), such as HO-1, which activates transcription through nuclear translocation of Nrf2 [51]. According to a study, Nrf2 can be activated by the PI3K/Akt pathway to prevent acute lung injury induced by high oxygen levels [52]. The reduction in oxidative stress may secondarily alleviate smooth muscle dysfunction and fibrosis. Cavernosum fibrosis is the primary pathology mechanism of ED, and high levels of oxidative stress and an inflammatory environment in tissues can cause the activation of the TGF-β1/p-Smad2/3/CTGF pro-fibrotic pathway and synthesis of collagen [53]. The PI3K/Akt/FOXO pathway is also involved in the regulation of fibrosis [54,55]. In the present study, GXN reduced NOX expression and ROS accumulation, increased antioxidant-associated markers including Nrf2-HO-1 and SOD, decreased MDA levels, and attenuated cavernous fibrosis. These findings are consistent with previous reports that natural products and vascular-protective agents can ameliorate diabetic complications through anti-oxidative and anti-fibrotic mechanisms.

In summary, our results indicate that DM leads to inhibition of the PI3K/Akt/FOXO1 pathway and increased apoptosis levels in both the corpus cavernosum and HCMECs. This condition causes oxidative stress activation in the corpus cavernosum, impairing the function of endothelial and smooth muscle cells, thereby increasing fibrosis levels. These complex molecular changes ultimately result in impaired erectile function. GXN supplementation alleviated DMED and was associated with activation of the PI3K/Akt/FOXO1 pathway, which may partly mediate its protective effects.

Several limitations should be acknowledged. First, this study used an STZ-induced type 1 diabetes model, whereas many clinical DMED patients have type 2 diabetes and complex metabolic comorbidities. Second, GXN is a multi-component formulation, and the active components responsible for the observed effects remain to be defined. Third, the evidence for PI3K/Akt/FOXO1 involvement is supportive rather than definitively causal: LY294002 was used only in HCMECs, and no in vivo pharmacological blockade or FOXO1 genetic manipulation was performed. Fourth, primary corpus cavernosum endothelial cells are an ideal in vitro model, but current primary isolation protocols remain technically immature, frequently resulting in poor purity, low yield, phenotypic instability, and a high risk of contamination by primary smooth muscle cells or fibroblasts. Therefore, we chose HCMECs as a reliable alternative with similar physiological characteristics and stable genomes. Future studies using primary cavernous endothelial cells, type 2 diabetes models, in vivo pathway inhibition, and FOXO1 gain- or loss-of-function approaches will be needed to establish stronger mechanistic causality and translational relevance.

## 5. Conclusions

GXN improved erectile function in STZ-induced type 1 diabetic rats and was associated with improved endothelial and smooth-muscle-related markers, reduced oxidative stress, attenuated cavernous fibrosis, and decreased apoptosis. Transcriptomic and protein-level analyses suggested that PI3K/Akt/FOXO1 signaling may be involved in the protective response to GXN, and in vitro PI3K inhibition partially reversed GXN-associated cellular protection. These findings provide supportive preclinical evidence for GXN as a potential therapeutic candidate for DMED. However, definitive in vivo mechanistic dependence on PI3K/Akt/FOXO1 signaling remains to be confirmed by pathway-blockade and genetic studies.

## Data Availability

The raw data supporting the conclusions of this article will be made available by the authors on request.

## Supplementary Materials

The following supporting information can be downloaded at https://www.mdpi.com/article/10.3390/biomedicines14081787/s1. Figure S1: In vivo workflow of this study; Figure S2: Differential enrichment pathways between the Control group and the DMED group. Table S1: The details of the antibodies used in the study.

## Abbreviations

The following abbreviations are used in this manuscript:

AP	Arterial pressure
AUC	Area under the curve
cGMP	Cyclic guanosine monophosphate
DMED	Diabetes mellitus-induced erectile dysfunction
GXN	Guanxinning tablet
HCMECs	Human cerebral microvascular endothelial cells
HG	High glucose
ICP	Intracavernous pressure
LG	Low glucose
MDA	Malondialdehyde
NADPH	Nicotinamide adenine dinucleotide phosphate
NO	Nitric oxide
ROS	Reactive oxygen species
SOD	Superoxide dismutase
STZ	Streptozotocin
TUNEL	Terminal deoxynucleotidyl transferase-mediated dUTP nick end labeling
