# Supplementary material for: Guanxinning Tablet Ameliorates Erectile Dysfunction in Diabetes Mellitus Rats with Involvement of PI3K/Akt/FOXO1 Signaling Pathway Modulation
Source: Biomedicines. 2026 Aug 7;14(8):1787. doi: 10.3390/biomedicines14081787 (PMC13509427; doi:10.3390/biomedicines14081787)
Supplement: Supplementary file 1 [file biomedicines-14-01787-s001.zip › Supplementary Figures.pdf]

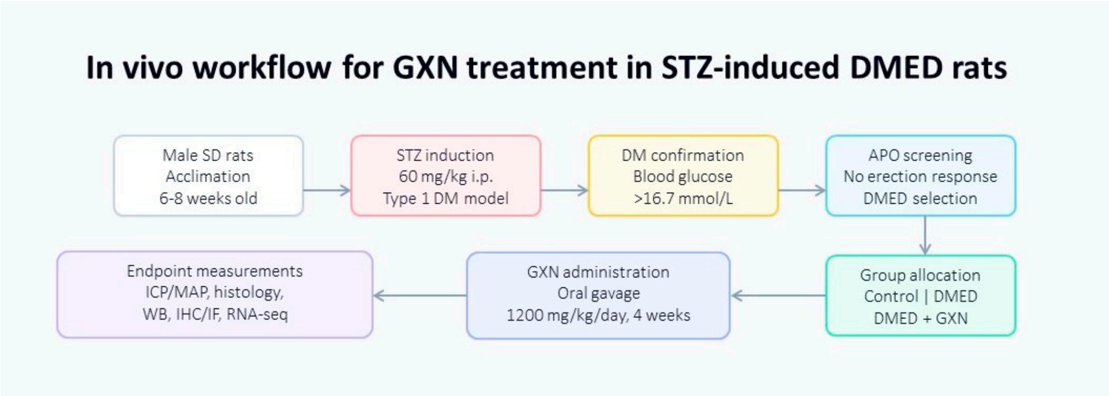

Supplementary Figure S1. In vivo workflow of this study.

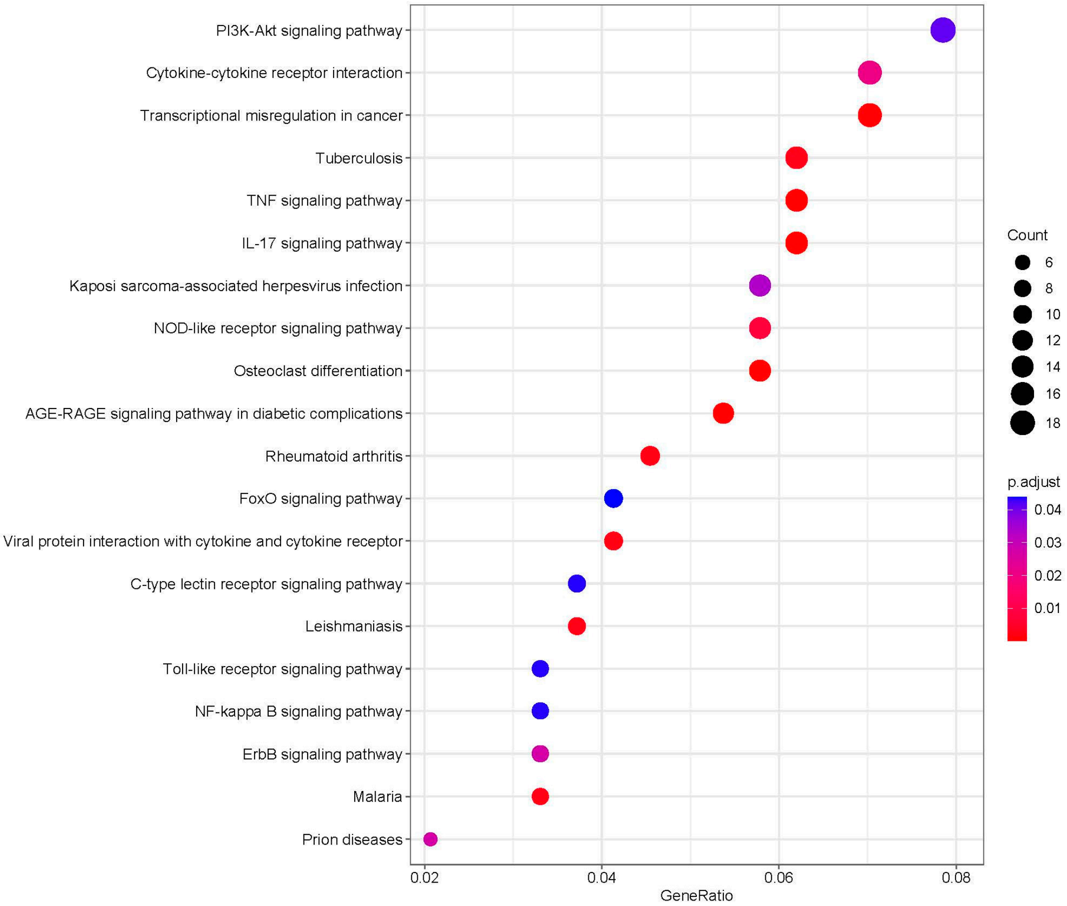

Supplementary Figure S2. Differential enrichment pathways between the Control group and the DMED group.
