# Supplementary material for: Guanxinning Tablet Ameliorates Erectile Dysfunction in Diabetes Mellitus Rats with Involvement of PI3K/Akt/FOXO1 Signaling Pathway Modulation
Source: Biomedicines. 2026 Aug 7;14(8):1787. doi: 10.3390/biomedicines14081787 (PMC13509427; doi:10.3390/biomedicines14081787)
Supplement: Supplementary file 1 [file biomedicines-14-01787-s001.zip › Supplementary Table S1.pdf]

Supplemental Table S1. The details of the antibodies used in the study

	Name	Abbreviation	Concentration	Source
WB	anti-eNOS	eNOS	1:500	ab184154; Abcam, Cambridge, MA, USA
	anti-p-eNOS	p-eNOS	1:500	9571; Cell Signaling Technology (CST), Danvers, MA, USA
	anti-RhoA	RhoA	1:1000	10749-1-AP; Proteintech, Wuhan, China
	anti-ROCK1	ROCK1	1:1000	21850-AP; Proteintech
	anti-ROCK2	ROCK2	1:1000	PB0428; Boster, Wuhan, China
	anti-Collagen I	Collagen I	1:1000	14695-1-AP; Proteintech
	anti-Collagen III	Collagen III	1:1000	68320-1-Ig; Proteintech
	anti-TGF-β1	TGF-β1	1:1000	21898-1-AP; Proteintech
	anti-Smad2/3	Smad2/3	1:1000	8685; CST
	anti-p-Smad2/3	p-Smad2/3	1:1000	8828; CST
	anti-CTGF	CTGF	1:1000	sc-101586; Santa Cruz Biotechnology, USA
	anti-α-SMA	α-SMA	1:1000	GB111364; Servicebio, Wuhan, China
	anti-Nrf2	Nrf2	1:500	AF0639; Affinity, USA
	anti-HO-1	HO-1	1:500	AF5393; Affinity
	anti-NOX1	NOX1	1:500	17772-1-AP; Proteintech, Wuhan, China
	anti-NOX2	NOX2	1:1000	19013-1-AP; Proteintech
	anti-NOX4	NOX4	1:1000	BM4135; Boster
	anti-p-PI3K	p-PI3K	1:1000	AP0854; Abclonal, Wuhan, China
	anti-PI3K	PI3K	1:1000	BA3558; Boster
	anti-p-AKT	p-AKT	1:1000	AP0637; Abclonal
	anti-AKT	AKT	1:1000	F0004; Selleck, Houston, TX, USA
	anti-p-FoxO1	p-FoxO1	1:1000	AP0172; Abclonal
	anti-FoxO1	FoxO1	1:1000	A2934; Abclonal
	anti-Bim	Bim	1:2000	A19702; Abclonal
	anti-Bax	Bax	1:1000	A19684; Abclonal
	anti-Bcl2	Bcl2	1:1000	A19693; Abclonal
	anti-Bad	Bad	1:1000	10435-1-AP; Proteintech
	anti-Caspase3	Caspase3	1:1000	AF6311; Affinity
	anti-cleaved-Caspase3	cleaved-Caspase3	1:500	AF7022; Affinity
	anti-β-actin	β-actin	1:1000	GB11001; Servicebio
IF	anti-CD31	CD31	1:500	GB11063; Servicebio

	anti-α-SMA	α-SMA	1:500	GB111364; Servicebio
	anti-FoxO1	FoxO1	1:500	A2934; Abclonal
IHC	anti-NOX2	NOX2	1:2000	BM4576; Boster
	anti-p-PI3K	p-PI3K	1:500	AP0854; Abclona
	anti-p-AKT	p-AKT	1:500	AP0637; Abclonal
	anti-p-FoxO1	p-FoxO1	1:500	AP0172; Abclonal
